# Whole Methylome Analysis by Ultra-Deep Sequencing Using Two-Base Encoding

**DOI:** 10.1371/journal.pone.0009320

**Published:** 2010-02-22

**Authors:** Christina A. Bormann Chung, Victoria L. Boyd, Kevin J. McKernan, Yutao Fu, Cinna Monighetti, Heather E. Peckham, Melissa Barker

**Affiliations:** 1 Life Technologies, Foster City, California, United States of America; 2 Life Technologies, Beverly, Massachusetts, United States of America; Memorial Sloan Kettering Cancer Center, United States of America

## Abstract

Methylation, the addition of methyl groups to cytosine (C), plays an important role in the regulation of gene expression in both normal and dysfunctional cells. During bisulfite conversion and subsequent PCR amplification, unmethylated Cs are converted into thymine (T), while methylated Cs will not be converted. Sequencing of this bisulfite-treated DNA permits the detection of methylation at specific sites. Through the introduction of next-generation sequencing technologies (NGS) simultaneous analysis of methylation motifs in multiple regions provides the opportunity for hypothesis-free study of the entire methylome. Here we present a whole methylome sequencing study that compares two different bisulfite conversion methods (in solution versus in gel), utilizing the high throughput of the SOLiD™ System. Advantages and disadvantages of the two different bisulfite conversion methods for constructing sequencing libraries are discussed. Furthermore, the application of the SOLiD™ bisulfite sequencing to larger and more complex genomes is shown with preliminary *in silico* created bisulfite converted reads.

## Introduction

The addition of methyl groups to cytosine (C) through DNA methyltransferases plays an important role in the regulation of human chromatin structure and gene expression. Methylation of C is involved in biological processes such as X chromosome inactivation, imprinting, embryogenesis, gametogenesis, and silencing of repetitive DNA elements in healthy and diseased cells. Thus, the study of DNA methylation can provide important insights into the regulation of cell differentiation, development, and diseases, such as cancer [1]. Most DNA methylation studies to date depend on pre-selection or enrichment of local genome areas through enzymatic approaches or the use of specific antibodies (MeDip) or DNA-binding proteins (e.g., MethylMiner) and subsequent Sanger sequencing technologies [2], [3]. However the biological importance and complex nature of DNA methylation has led to an increased interest in studying this phenomenon on a global approach, i.e., a methylation profile of the whole genome or “methylome”.

**Figure 1:**
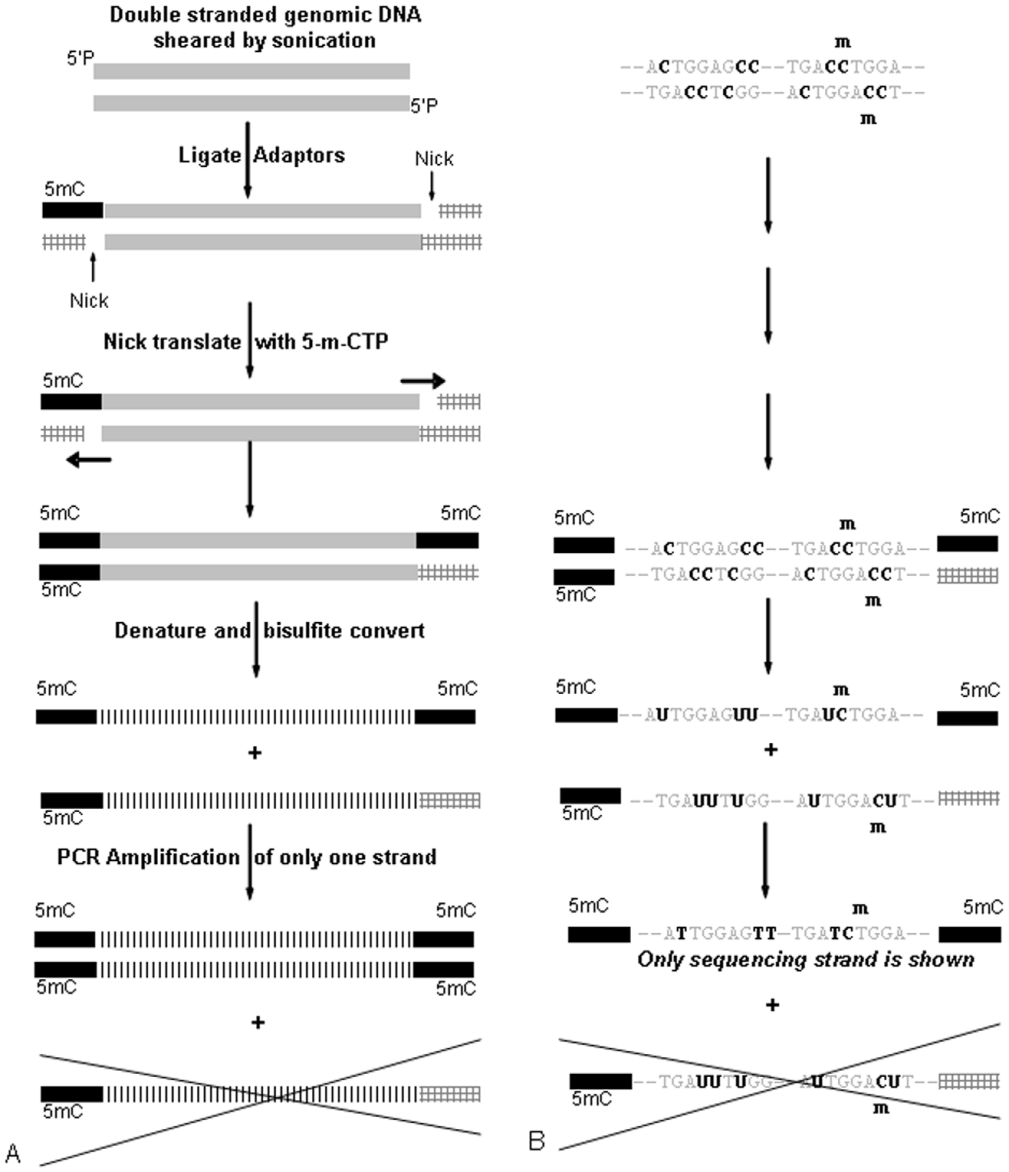
Library construction to protect the adapter sequence from bisulfite conversion. Genomic DNA (5 ug) was sheared by sonication and end-repaired to yield 5′-phosphorylated (5′P) blunt ends. Two double-stranded oligonucleotide adaptors, having only one preselected oligonucleotide protected by 5mC (5mC/black) against bisulfite conversion, were ligated to the DNA fragments. After nick-translation with 5mC-dNTP one adaptor consists of two fully 5mC-protected oligonucleotides, whereas the other adaptor still contains one oligonucleotide with unprotected regular Cs (Fig. 1A). Following a size selection to 175–225 bp on an agarose gel, equal amounts of DNA (240 ng) were used for bisulfite conversion in solution and in gel, respectively. During bisulfite conversion, the DNA is denatured and due to only three 5mC-protected adaptor strands, the fourth adaptor strand was bisulfite converted thus changing the sequence by altering C to U. During PCR amplification (**scheme B**) with regular four-base primers (A, G, C, and T) complement to the library adaptors, only one of the fragments was amplified. 5′P  =  5′-phosphorylated blunt ends, 5mC  =  5-methylcytosine.

Bisulfite sequencing is widely used for DNA methylation profiling, because of its accuracy and its ability to provide information about the methylation status of C independent of the genomic location or sequence context [4]. Prior to sequencing, the DNA is bisulfite treated, which converts unmethylated Cs to uracil (U), while 5-methylcytosine (5mC) remain unchanged. During subsequent PCR amplification, C to thymine (T) changes will be introduced into the sequence at non-methylated C sites and Cs remain at 5mC sites (Fig. 1B), thus allowing an exact interrogation of all possible methylation sites in the genomic sequence. Next-generation sequencing technologies (NGS) provide an ideal tool for DNA methylation profiling, due to their massively parallel sequencing capability, which provides a huge amount of data in a relatively short amount of time at minimal cost per base compared to Sanger sequencing technologies. Whereas several studies have been published that use NGS to perform DNA methylation profiling following an enrichment technique [2], [5]–[9], only one other study to our knowledge has been published studying a whole methylome with bisulfite sequencing [10].

Here we present the first whole methylome bisulfite sequencing study using the SOLiD™ (Sequencing by Oligonucleotide Ligation and Detection) platform. This technology differs from other NGS technologies by the interrogation of two bases at a time by ligation chemistry, and detection of one of four colors associated with those specific two bases (for more details on this technology and color sequencing, see [11]–[14]).

We also show the comparison of two bisulfite conversion methods–performing bisulfite conversion in solution versus in a polyacrylamide gel. The SOLiD sequencing reported here was performed with bacterial libraries that were prepared similar to our previous publication [15], but with some differences that were informative.

## Results and Discussion

### Library Construction and Bisulfite Conversion in Solution versus in Gel

Two bisulfite-converted libraries of *Escherichia coli* (*E. coli*) DH10B were constructed, and the bisulfite conversion was performed in solution (bis-sol) or in a polyacrylamide gel (bis-gel) as previously described [15] (see Figure 1 for outline). Equal amounts of DNA (240 ng) were used for both bisulfite conversion methods to compare the efficiency of each method. Both bis-sol and bis-gel amplified equally well with 12 PCR cycles yielding 2.19 ng/ul and 2.39 ng/ul of DNA, respectively, thus showing that both conversion methods result in libraries of equal quantity. Apparently, DNA loss is not as high as previously anticipated during in solution bisulfite conversion. However, there are less experimental steps during in-gel bisulfite conversion, shortening the hands-on time in the lab. On the other hand, the in-gel method is limited by the DNA input, because the thin polyacrylamide gel has a low DNA capacity. Therefore, the in solution method is preferred for larger DNA input (>2 ug starting material), while the in gel method is ideal for low amounts of starting material. As shown previously [15], 50 ng and even 5 ng can be bisulfite converted successfully in a gel with 15 cycles and 22 cycles of PCR amplification. This would correspond to about 500 ng or 50 ng of starting material, respectively, if losses are assumed to be the same as in the experiment described here. Loss of library molecules for NGS applications is apparent for other bisulfite conversion protocols [10], [16] as evidenced by the need for higher DNA input (5 ug) and higher required number of cycles for amplification (18 cycles). A lower number of PCR cycles during library construction is desired to prevent the introduction of PCR biases and consequently reduced complexity of libraries.

### SOLiD Sequencing

The two libraries, bis-sol and bis-gel, were amplified on magnetic beads by emulsion PCR (ePCR) according to standard SOLiD protocols, with the exception that additional dATP and dTTP was added to the aqueous ePCR phase to compensate for the low complexity of the bisulfite converted libraries. A 5% increase in the concentration of dATP and dTTP was sufficient to improve ePCR yields and is within the expected variable caused by hydrolysis of the dNTPs to di- and monophosphate. The slight change in the dNTP ratio is below that used to cause mutagenesis [17]. Additionally, bisulfite-SOLiD sequencing data provided excellent reference matching, similar to the non-bisulfite treated DH10B data. Each library was then sequenced to 50 base pairs on two quarters of a slide with SOLiD 3.0 chemistry. After bisulfite conversion, the sense and antisense (+/−) DNA strands are no longer complementary: non-methylated Cs in both the + and the − strand will appear in the SOLiD reads as T, whereas only methylated Cs appear as Cs (Fig. 1B). Thus, two bisulfite converted reference sequences were created *in silico* from DH10B by replacing all Cs with Ts for the + and the − strand, respectively (+/− bisulfite converted reference). As a control, regular non-bisulfite-converted DH10B genomic sequence was used (normal reference). The SOLiD reads from the two libraries were mapped towards all three reference sequences allowing a maximum of five mismatches, using the SOLiD™ System Analysis Pipeline Tool.

**Figure 2:**
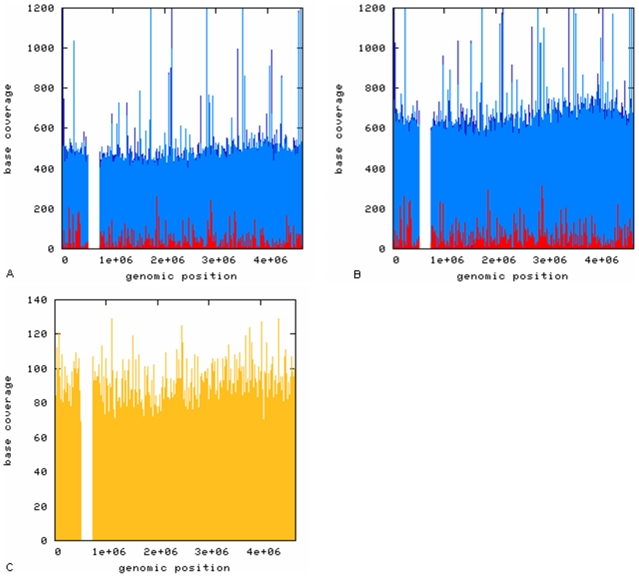
Comparison of the sequencing coverage for bis-sol, bis gel and a non-bisulfite converted DH10B sequencing run. Plots A (**bis-sol**) and B (**bis gel**) show the coverage of sequencing reads against the +/− bisulfite converted references (dark/light blue) and the normal reference (red). As a comparison, plot C shows the coverage of a regular non-bisulfited DH10B sequencing run matched towards the normal reference (yellow) (Data from SOLiD software communtiy web-site). The missing coverage in all three plots corresponds to a 133 kb perfect repeat in tandem. Since reads will match to both repeats, they will not show up under the unique matches.

Due to the design of the library construction, only one DNA-fragment strand had fully 5mC-protected Adapter sequences and was therefore amplified during large-scale library PCR (Fig. 1). Consequently, reverse-complement reads are non-existent and matches to the reverse-complement of the bisulfite converted reference are invalid. During the matching pipeline in the SOLiD™ System Analysis Pipeline Tool, reads are automatically mapped to both the forward and reverse-complement sequence of the reference. Therefore, reads that were erroneously mapped to the reverse-complement strand of the + or − bisulfite converted reference were removed from the mapping file, and the new file was put through the Analysis Pipeline Tool again to obtain the correct mapping statistics. Through comparison of the first (including matches to the reverse-complement strand) and the second mapping statistics, the amount of mismapped reads was determined. Only 0.010–0.015% of total matches were mismapped to the reverse-complement strand (Table 1). Investigating the mismapped reads for library bis-sol, − bisulfite reference further, showed that 0.00003% of reads were expected mismatches due to bisulfite conversion, 0.002% were mismapped due to CCWGG motifs in the reads, 0.003% were mismapped due to incomplete bisulfite conversion, and 0.01% were mismapped due to instrument errors or contamination (resulting in 0.015% of total mismapped reads). The mapping overall showed to be very accurate (>99.98%) and comparable to non-bisulfite SOLiD sequencing. The total number of matches to the bisulfite converted reference (as shown in table 2) can be obtained by adding up all reads mapped to both the + and − bisuflite converted reference. In both libraries (bis-sol and bis-gel) ∼60% of total reads could be matched uniquely ( =  each read maps to one single position on the reference sequence) to the +/− bisulfite converted reference sequence. This number is comparable to unique matching statistics of sequence reads from regular non-bisulfite converted DH10B libraries (SOLiD™ System E. coli DH10B Fragment Data Set, http://solidsoftwaretools.com/gf/project/dh10bfrag/). As expected, the bisulfite-converted libraries (bis-sol and bis-gel) did not map well against the normal reference (Table 2). Less than 0.1% of sequencing reads were mapped uniquely to this reference. This difference in unique matching (towards +/− bisulfite converted or normal reference) is also illustrated in Figures 2A and B, which show a coverage plot of sequencing reads from both libraries against the three reference sequences. Sequencing reads of both libraries covered the + and − bisulfite converted references more than 270 times. The coverage plots (Figures 2A and B), reveal one large non-covered region. This region contains a 113 kb region that is exactly duplicated in tandem [18]. Thus, sequencing reads will map to both duplications and do not appear as uniquely mapped reads. A coverage plot of regular non-bisulfite converted DH10B sequencing reads (Figure 2C, sequencing data from SOLiD™ System *E. coli* DH10B Fragment Data Set, http://solidsoftwaretools.com/gf/project/dh10bfrag/) shows the same region uncovered.

**Table 1:** Percent of mismatches to the reverse complement strand of the bisulfite converted reference.

		+ bisulfite converted Reference		− bisulfite converted Reference	
		Count	%	Count	%
**bis-sol**	Total matches	21,197,732		21,227,698	
	Mismatches to the reverse complement	3,049	0.014	3,129	0.015
**bis-gel**	Total matches	28,901,701		29,019,613	
	Mismatches to the reverse complement	3,100	0.011	3,013	0.010

According to the library construction design, bisulfite converted reads should not map to the reverse complement of the +/− bisulfited reference.

Sequencing reads of the two libraries (bis-sol and bis-gel) were first mapped to both strands of each reference. Invalid matches to the reverse complement strand were then filtered out and counted.

**Table 2:** Matching statistics of libraries bis-sol and bis-gel against bisulfite converted and normal reference.

		+/− bisulfite converted Reference		Normal Reference	
		Count	%	Count	%
**bis-sol**	Beads found	74,366,093		74,366,093	
	Uniquely placed beads (≤5 mismatches)	42,419,252	57.04	35,389	0.05
	Bases not uniquely covered	332,578	7.10	4,087,762	87.22
**bis-gel**	Beads found	89,599,880		89,599,880	
	Uniquely placed beads (≤5 mismatches)	57,915,201	64.64	68,581	0.08
	Bases not uniquely covered	330,772	7.06	3,436,445	73.32

**Figure 3:**
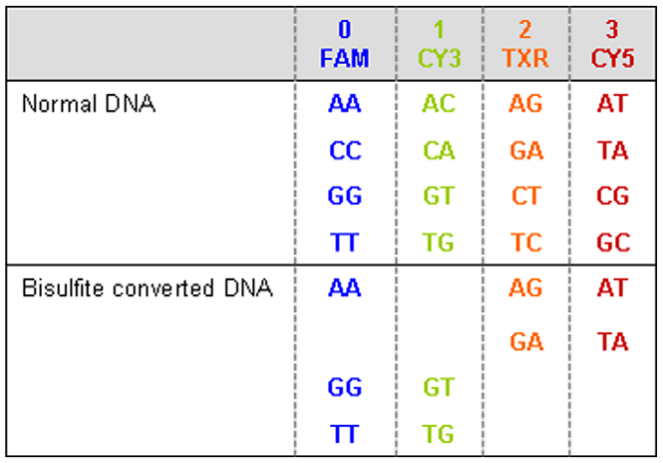
Sequencing of normal versus bisulfite converted DNA using two-base encoding. During bisulfite conversion unmethylated cytosine (C) gets converted to thimine (T), thus reducing the sequence into predominantly three bases (adenine (A), guanine (G), and T). During color space sequencing, two bases are interrogated at the same time, resulting in one color being recorded. Thus sequencing a reduced complex sequence, such as bisulfite converted DNA, still results into all four colors being used during sequencing. FAM, CY3, TXR, CY5: Fluorescent labels used for color space sequencing.

The conversion of the genome from a 4-base alphabet to a 3-base alphabet lengthens the number of bases required for unique alignment. This reduced complexity in base space increases the number of non-unique sequence matches to the *in silico* bisulfite converted genome. SOLiD sequencing is based on two-base interrogation during color-space sequencing, which retains all four sequencing colors, even when sequencing a genome lacking several of the 16 possible two-base combinations, such as in bisulfite sequencing (Figure 3). The assembly of the genome as dinucleotide units (color space) increases the ability to uniquely map in color space relative to base space [13]. The purpose of bisulfite sequencing is to identify methylated CpGs which manifest in short read sequences as mismatches. With perfectly aligned reads that contain methylated motifs, both base space and color space will provide unique mapping. However, *in silico* calculations using the bisulfite-converted human genome that permits mismatches in reads containing methylated CpGs show a 5% increase in unique mapping for 25 bp reads (2 mismatches) and 50 bp reads (5 mismatches) in chromosome 20. This color space advantage increases to 40% over base space when mapping the *in silico* created bisulfite converted 25 bp reads (with 2 mismatches) for the entire human genome. This increase in unique mapping is expected to extend to the mapping of longer reads (50 bp, 5 mismatches) in color space, as shown through a sampling approach with chromosome 17. Aligning this chromosome against the whole genome reveals an advantage of mapping in color space by 42% for 25 bp reads (2 mismatches) and 8% for 50 bp reads (5 mismatches) in terms of percentages of uniquely aligned portions.

### Identification of Methylated CCmWGG Sites (W  =  either A or T)

Two DNA methylases are present in *E. coli*, *dam* and *dcm*. While *dam* methylates adenine (A), *dcm* methylates the second C residue in the motif CCWGG to form 5mC [19]–[20]. Therefore, non-bisulfite converted Cs, which correspond to methylated Cs, should only be present in this motif. In order to find those methylated Cs, the uniquely mapped reads (to the + and − bisulfite converted references) were first analyzed using the SNP pipeline of the SOLiD™ System Analysis Pipeline Tool. Non-bisulfite converted Cs will show up in this analysis as a valid SNP T → C (+ or − bisulfite converted reference → sequencing read). Thus, all positions of T → C SNPs were matched to the location of CCWGG sites to determine eligibility of a non-bisulfite converted C to be methylated. All other T → C SNPs are therefore incomplete bisulfite converted Cs. Only between 0.001–0.003% of Cs fell into the later category (Table 3), thus showing that bisulfite conversion during library construction in-gel and in-solution were both greater than 99.99%.

**Table 3:** Methylation status of CCWGG sites and bisulfite conversion efficiency in bis-sol and bis-gel.

		+ bisulfite converted Reference		− bisulfite converted Reference	
		Count	%	Count	%
**bis-sol**	CCWGG sites covered	11,295		11,292	
	CCWGG	0	0.00	0	0.00
	CCmWGG	11,241	99.52	11,236	99.50
	CYWGG	55	0.49	56	0.50
	Total C	1,190,995		1,188,905	
	unconverted C	32	0.003	23	0.002
**bis-gel**	CCWGG sites covered	11,304		11,302	
	CCWGG	0	0.00	0	0.00
	CCmWGG	11,252	99.54	11,248	99.52
	CYWGG	52	0.46	54	0.48
	Total C	1,190,995		1,188,905	
	unconverted C	19	0.002	16	0.001

Fully CCmWGG and partially methylated CYWGG sites were discovered by comparing the sequencing reads against the +/− bisulfite converted reference. Methylated Cs will show up as a SNP conversion T → C (bisulfite converted reference → sequence read) within the CCWGG motif. A T → C SNP outside this motif indicates an incomplete bisulfite conversion (unconverted C). Comparing this number with the total number of Cs present in the DH10B genome, indicates the bisulfite conversion efficiency of the library protocol.

**Figure 4:**
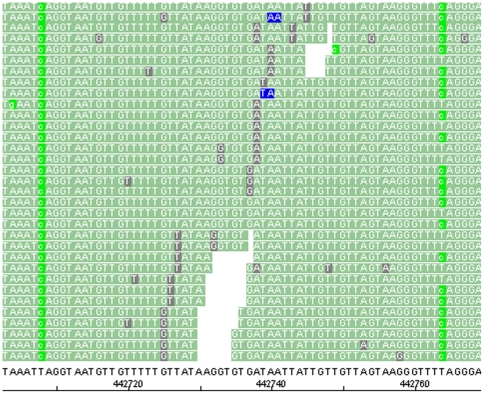
Visualization of methylated CCmWGG and hemimethyalted CYWGG sites with the SOLiD™ System Alignment Browser. One fully methylated CCmWGG sites (seen at the left) and one partially methylated CYWGG site (right) are shown. Methylated Cs show up as a T → C (bisulfite converted reference → sequencing read) SNP conversion (light green), while non-methylated Cs show up as a T in the bisulfite converted context and match to the bisulfite converted reference. light green  =  valid adjacent mismatch  =  SNP, blue  =  invalid adjacent mismatch, grey  =  isolated mismatch (for details on mapping in color space and definition of valid versus invalid mismatches, please refer to 11).

Of the expected 12,174 CCWGG motifs present in the DH10B genome sequence, ∼11,300 or ∼92.8% motifs were covered with both bis-sol and bis-gel libraries (Table 3). Over 99.5% of those covered CCWGG sites were methylated (CCmWGG) and the remaining 0.5% were partially methylated (CYWGG) (Figure 4). The complete or nearly complete methylation of all CCWGG sites was expected, since *E. coli* has a repair system that preserves methylated *dcm* sites [19]. Each base of the genome was sequenced multiple times (∼300-fold coverage), which permitted easy identification of partially methylated sites. A site was called partially methylated, if (1) T ( =  non-methylated) was called at least 25% of the time or if (2) T was called at least 14% and the same genomic position was partially methylated after criteria 1 in the corresponding library (bis-sol or bis-gel). All 52 or 54 (+ or − bisulfite converted reference, respectively) CYWGG sites that were found in the bis-gel library were also present in the bis-sol library. In total 44 CYWGG sites were present in both libraries (bis-sol and bis-gel) and on both strands (+/− bisulfite converted reference). The biological significance of partially methylated sites in DH10B is unknown and requires further investigation. However, the detection of those sites by SOLiD sequencing demonstrates how deep-sequencing of the whole methylome and determination of the methylation status at specific sites can be achieved by next-generation sequencing technologies.

In order to investigate, how a lower mismatch number might affect the detection sensitivity for methylation and incomplete bisulfite conversion due to higher reference bias, a genotyping approach was used to locate all reads that match a CCWGG motif. Read counts for each motif were then adjusted by using different number of mismatches (2, 3, 4, and 5 mismatches, respectively) (Table S1). Beyond the slight gains by increasing the number of mismatches from 2 to 3 and from 3 to 4, the high coverage of SOLiD data practically eliminates any reference bias due to low mismatch numbers. All CCWGG motifs in DH10B are separated far enough from each other, so that at least one local 50 bp read covering each Cm can in theory be correctly matched to the bisulfite converted genome, using at least 4 mismatches. There are 57 motifs that can not be queried *in silico* by any 50 bp read at 3 mismatches, and 61 motifs at 2 mismatches, which results in a 99.5% theoretical coverage of all CCWGG motifs. However, the human genome is more difficult to query for methylated CpG motifs, due to clustered CpG-islands. These non-separable islands need to be mapped to unconverted reference sequences in order to ensure correct matching.

In conclusion, two different bisulfite conversion methods were compared using a 5mC-protected adaptor protocol to construct libraries of *E. coli* strain DH10B. Regardless of the bisulfite conversion method, excellent mapping statistics to *in silico* bisulfite-converted references were obtained and methylated CCWGG sites were identified. Thus, both library construction methods are equally well-suited for whole methylome sequencing.

Furthermore, this study shows that SOLiD-bisulfite sequencing is sensitive enough to identify partially methylated sites. The ability to detect the relatively rare methylation event at a single site has to date been hampered by the limitations of the tools traditionally used to study methylation. This new level of detection will be an advantage in many applications of bisulfite sequencing in which copy number variation of a specific methylation motif is of biological importance. Additionally, preliminary *in silico* computations indicate an advantage of bisulfite color versus base space sequencing in more complex genomes, such as human. However, only future bisulfite sequencing experiments of a human genome on NGS platforms, will give a definite answer to this question.

## Materials and Methods

### Library Construction (Figure 1A)

The protocol is based upon the “SOLiD™ System Fragment Library Preparation: Higher Input (2–20 ug) or Higher-Complexity DNA” protocol of the Applied Biosystems SOLiD™ System v2.0 User Guide (Applied Biosystems, Foster City, CA, USA). Bacterial DH10B gDNA (Lofstrand Labs Limited, Gaithersburg, MD, USA) (5 µg) was sheared into fragments in a 13×65 mm borosilicate tube (Covaris, Woburn, MA, USA) in 500 µL 10 mM Tris, pH 8.0, plus 5% w/v 2-micron borosilicate glass dry spheres (Duke Scientific Corporation, Fremont, CA, USA), using a Covaris S2 (Covaris, Woburn, MA, USA) (shearing conditions: cycle no. 10, bath temperature 5°C, Mode: power tracking, duty cycle 20%, intensity 10, cycles/burst 1000, time 60 sec). The sheared DNA was subsequently purified with the MinElute Reaction Cleanup Kit (Qiagen, Valencia, CA, USA) according to the manufacturer's instructions using buffer ERC and eluting the DNA off the columns using two times 15 uL buffer EB. The DNA was then quantitated using a NanoDrop ND 1000 Spectrophotometer (Thermo Fisher Scientific, Waltham, MA, USA). In order to repair damaged DNA ends and obtain 5′-phosphorylated blunt-ends (5′P), the fragments were end-repaired using the End-It DNA End-Repair Kit (Epicentre Biotechnologies, Madison, WI, USA) according to the manufacturer's instructions and incubated at room temperature for 30 minutes. The enzymatic reaction was purified using the MinElute Reaction Cleanup Kit and the DNA recovered with two 20 ul buffer EB elutions, then quantified as described above. Adaptors used in this protocol deviated from the standard SOLiD fragment library protocol in that the top strand P1-A of the double-stranded P1 adaptor was synthesized using 5mC in place of C in order to protect the adaptor from modification during bisulfite conversion (for details and sequences see [15]) whereas double-stranded adaptor P2 was identical to the adaptor used in the standard protocol. The adaptors were ligated to the end-repaired DNA fragments in a 30∶1 molar ratio for 10 min at room temperature using the Quick Ligation Kit (New England BioLabs, Beverly, MA, USA) (1x quick ligase reaction buffer, 1 uL quick ligase/40 µL reaction volume). The ligation reaction was purified using Agencourt AMPure beads (Agencourt Bioscience Corporation, Beverly, MA, USA) in order to exclude any remaining 68 bp adaptor-dimers. For this, 1.8 volumes of AMPure beads were added to the ligation reaction and incubated with rotation for 5 minutes at room temperature. Then the beads were placed on a magnetic stand and the supernatant was discarded. The beads were washed three times with 70% ethanol, air dried, and DNA was eluted off the beads by adding 35 µL 10 mM Tris, pH 8.0. During the adaptor ligation only the 5′-3′ strand of the adaptor ligated to the 5′P ends of the DNA fragments. After the DNA purification the 3′-5′ adaptor strands were filled in by nick-translation using a dNTP solution, containing 5m-dCTP instead of dCTP as previously described [15]. The nick translation reaction was carried out for 30 minutes at 16°C using DNA Polymerase I (New England BioLabs, Beverly, MA, USA) (1x NEB buffer 2, 2 mM 5mC-dNTP, 0.25 U/µl DNA Polymerase I). The enzymatic reaction was purified with the MinElute Reaction Cleanup Kit using 20 µL buffer EB to elute the DNA off the column and quantitated as described above.

The nick-translated DNA was then size selected to 175–225 bp on a 3% agarose gel (BioRad Laboratories, Hercules, CA, USA). In order to purify the DNA from the gel, the MinElute Gel Extraction Kit (Qiagen, Valencia, CA, USA) was used by adding six volumes of buffer QG to the gel pieces and vortexing the mixture until the gel was dissolved (about 5 min). This solution was then applied to the MinElute columns and washed according to manufacturer's instructions. The DNA was eluted off the columns by applying 25 µL buffer EB and quantitated as described above.

A 240 ng aliquot of the size-selected DNA was bisulfite converted in solution as previously described [15]. An equal aliquot was run into a 6% cross-linked Retardation Gel (Invitrogen, Carlsbad, CA, USA). The DNA band was cut from the gel and bisulfite converted within the gel as described previously [15]. The bisulfite-converted DNA from both bisulfite conversion methods was PCR amplified in three 100 µl reactions using 1x Platinum PCR Supermix (Invitrogen, Carlsbad, CA, USA), 1 µM primer 1 & 1 µM primer 2 (Standard SOLiD library PCR primers, Applied Biosystems, Foster City, CA, USA), 0.025 U/µL AmpliTaq DNA Polymerase, LD (Applied Biosystems, Foster City, CA, USA). The cycling conditions were as follows: 95°C for 5 min; 12 cycles of 95°C for 15 sec, 62°C for 15 sec, & 70°C for 1 min; 70°C for 5 min. After PCR amplification the in gel bisulfite converted library (bis-gel) was applied to a 0.45 µm filter NanoSep column (Pall Life Sciences, East Hills, NY, USA) and centrifuged for 5 min at 10,000×g to remove gel pieces from the library solution. The bis-gel and the bis-sol libraries were then purified using AMPure beads as described above and DNA was eluted from the beads in 20 µL 10 mM Tris, pH 8.0. The two libraries were finally quantitated using the 2100 Bioanalyzer with a DNA 1000 Chip (Agilent Technologies, Santa Clara, CA, USA) and using the Qubit fluorometer with the Quant-it dsDNA HS Kit (Invitrogen, Carlsbad, CA, USA).

### Templated Bead Preparation

Emulsion PCR (ePCR) was performed according to standard Applied Biosystems SOLiD™ 3 System: Templated Bead Preparation Guide, with the exception that extra dATP and dTTP (Invitrogen, Carlsbad, CA, USA) were added to the aqueous phase to compensate for the AT-rich template due to bisulfite conversion. The composition of the aqueous phase was as follows: 1x PCR buffer, 14 mM dNTP, 0.7 mM dATP, 0.7 mM dTTP, 25 mM MgCl_2_, 40 nM ePCR primer P1, 3 µM ePCR primer P2, 0.54 U/µL AmpliTaq Gold DNA polymerase. The aqueous phase was then introduced to a whirling oil phase in an ULTRA-TURRAX® Turbo Drive (IKA, Staufen, Germany) to create a water-in-oil emulsion. This emulsion was transferred to a 96-well plate and thermocycled using the recommended PCR conditions. After PCR amplification, emulsions were broken using butanol, the beads were washed, enriched, and terminal transferased before quantification and deposition onto a slide for sequencing according to manufacturer's instructions.

### Sequencing of Templated Beads

Templated beads were deposited onto two slide quadrants per sample and sequencing was carried out to 50 bases using SOLiD v3.0 chemistry and manufacturer's instructions.

### Data Analysis

Two bisulfite converted references (+ and − strands) were created by replacing *in silico* all Cs to Ts in the sense (+) and antisense (−) strands of the DH10B genome (GenBank accession CP000948), respectively. Sequencing reads were aligned to the + and − bisulfite converted references and the DH10B genome (normal reference) using the SOLiD™ System Analysis Pipeline Tool (http://solidsoftwaretools.com/gf/project/corona/), allowing a maximum of five mismatches per read. Matches to the reverse complement of the + and − bisulfite converted references were discarded, since only one strand was amplified during library construction and thus matches to the reverse complement are mismatches. SNPs in SOLiD™ datasets were identified using the SOLiD™ System Analysis Pipeline Tool. Non-converted Cs were identified and matched with the respective positions of possible methylation sites (motif CCWGG) in the DH10B genome.

## Supporting Information

Table S1 Comparison of different number of mismatches on detection sensitivity for methylation and incomplete bisulfite conversion.
(0.05 MB DOC)
