# Supplementary material for: Whole Methylome Analysis by Ultra-Deep Sequencing Using Two-Base Encoding
Source: PLoS One. 2010 Feb 22;5(2):e9320. doi: 10.1371/journal.pone.0009320 (PMC2825269; doi:10.1371/journal.pone.0009320)
Supplement: Table S1 — Comparison of different number of mismatches on detection sensitivity for methylation and incomplete bisulfite conversion. (0.05 MB DOC) [file pone.0009320.s001.doc]

**Table S1: Comparison of different number of mismatches on detection sensitivity for methylation and incomplete bisulfite conversion.**

A genotyping approach was used to locate all reads that match a CCWGG motif or called T->C SNPs at 5 mismatches. SNP calls were then adjusted by using different number of mismatches (2, 3, 4, and 5 mismatches, respectively, separated by ‘/’ in the table below).

|  |  | **+ bisulfite converted Reference** | | **- bisulfite converted Reference** | |
| --- | --- | --- | --- | --- | --- |
|  |  | **Count** | **%** | **Count** | **%** |
| **bis-sol** | CCWGG sites covered | 11,091/11,226/11,289/11,310 |  | 11,103/11,231/11,276/11,299 |  |
|  | CCWGG | 133/68/18/1 | 1.20/0.61/0.16/0.01 | 110/63/12/1 | 0.99/0.56/0.11/0.01 |
|  | CCmWGG sites | 10,011/10,803/11,135/11,252 | 90.3/96.2/98.6/99.5 | 10,101/10,793/11,153/11,237 | 91.0/96.1/98.9/99.5 |
|  | CYWGG | 672/279/117/54 | 6.1/2.5/1.0/0.5 | 670/298/95/54 | 6.0/2.7/0.8/0.5 |
|  | Total C | 1,190,995 |  | 1,188,905 |  |
|  | Under-converted C | 6/22/45/64 | 0.001/0.002/0.004/0.005 | 6/12/22/45 | 0.001/0.001/0.002/0.004 |
| **bis-gel** | CCWGG sites covered | 11,185/11,268/11,306/11,314 |  | 11,198/11,267/11,296/11,302 |  |
|  | CCWGG | 97/58/5/1 | 0.87/0.51/0.04/0.01 | 93/59/7/1 | 0.83/0.52/0.06/0.01 |
|  | CCmWGG sites | 10,508/10,944/11,207/11,265 | 94.0/97.1/99.1/99.6 | 10,575/10,984/11,211/11,265 | 94.4/97.5/99.3/99.7 |
|  | CYWGG | 460/219/79/44 | 4.1/1.9/0.7/0.4 | 400/171/65/34 | 3.6/1.5/0.6/0.3 |
|  | Total C | 1,190,995 |  | 1,188,905 |  |
|  | Under-converted C | 6/15/36/53 | 0.001/0.001/0.003/0.004 | 4/16/27/49 | 0.000/0.001/0.002/0.004 |
